# Supplementary material for: CD4+/CD45RO+: A Potential Biomarker of the Clinical Response to Glatiramer Acetate
Source: Cells. 2019 May 15;8(5):456. doi: 10.3390/cells8050456 (PMC6562382; doi:10.3390/cells8050456)
Supplement: Supplementary file 1 [file cells-08-00456-s001.pdf]

cells

Article

CD4+/CD45RO+: A Potential Biomarker of the Clinical Response to Glatiramer Acetate

Martin Vališ [1], Lukáš Sobíšek [1], Oldřich Vyšata [1], Blanka Klímová [1], Ctirad Andrýs [2], Doris Vokurková [2], Jiří Masopust [1] and Zbyšek Pavelek [1,*]

1. Department of Neurology, Faculty of Medicine and University Hospital Hradec Králové, Charles University in Prague, Sokolská 581, 500 05 Hradec Králové, Czech Republic; martin.valis@fnhk.cz (M.V.); lukas.sobisek@yahoo.com (L.S.); oldrich.vysata@fnhk.cz (O.V.); Blanka.Klimova@uhk.cz (B.K.); jiri.masopust@fnhk.cz (J.M.)
2. Department of Clinical Immunology and Allergology, University Hospital Hradec Králové, Sokolská 581, 500 05 Hradec Králové, Czech Republic; ctirad.andrys@fnhk.cz (C.A.); doris.vokurkova@fnhk.cz (D.V.)

* Correspondence: zbysekpavelek@email.cz; Tel. +420495835251; Fax: +420495835216

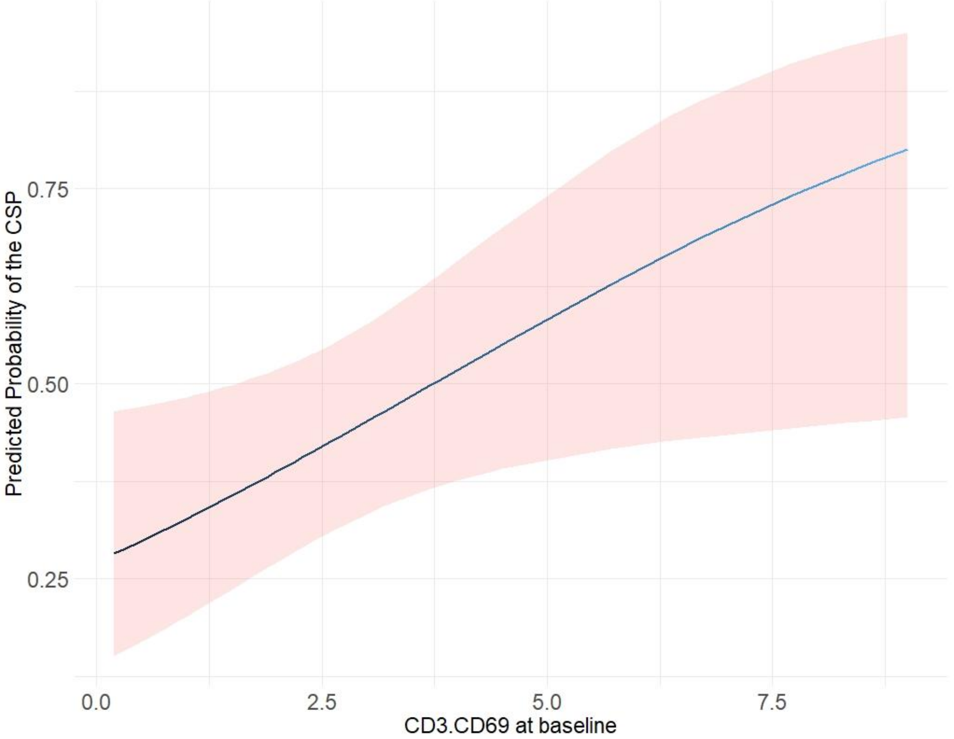

Supplementary Figure 1. Predicted probability of the confirmed sustained progression (CSP) (CD3+/CD69+ at baseline). Legend: The figure shows the predicted probability of the CSP (on vertical axis) for the range of measured values (on the horizontal axis) of a lymphocyte parameter (labelled at the bottom of the figure). The blue curve depicts the point estimate of the probability and the pink area represents its 95% confidence interval.

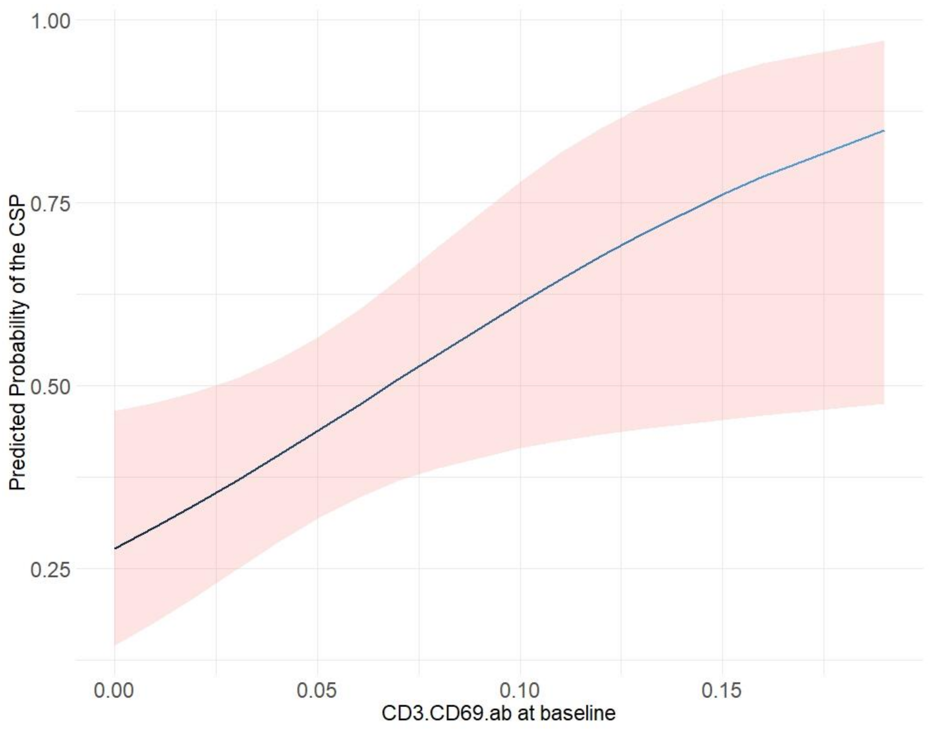

Supplementary Figure 2. Predicted probability of the CSP (CD3+/CD69+ab at baseline). Legend: The figure shows the predicted probability of the CSP (on vertical axis) for the range of measured values (on the horizontal axis) of a lymphocyte parameter (labelled at the bottom of the figure). The blue curve depicts the point estimate of the probability and the pink area represents its 95% confidence interval.

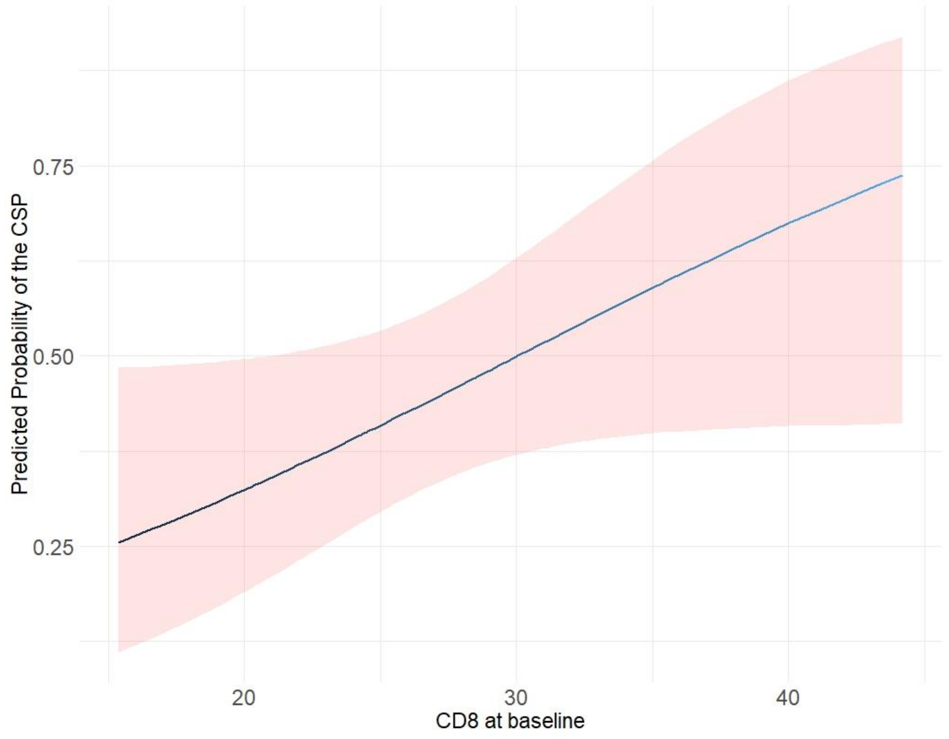

Supplementary Figure 3. Predicted probability of the CSP (CD4+/CD45RA+ at baseline). Legend: The figure shows the predicted probability of the CSP (on vertical axis) for the range of measured values (on the horizontal axis) of a lymphocyte parameter (labelled at the bottom of the figure). The blue

curve depicts the point estimate of the probability and the pink area represents its 95% confidence interval.

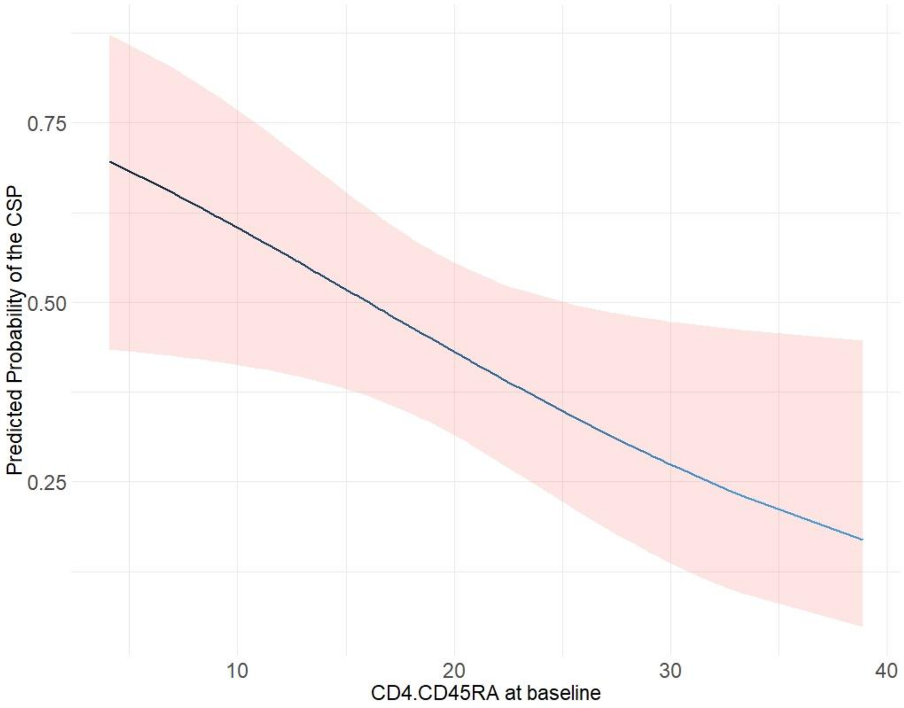

Supplementary Figure 4. Predicted probability of the CSP (CD4+/CD45RO+ at baseline). Legend: The figure shows the predicted probability of the CSP (on vertical axis) for the range of measured values (on the horizontal axis) of a lymphocyte parameter (labelled at the bottom of the figure). The blue curve depicts the point estimate of the probability and the pink area represents its 95% confidence interval.

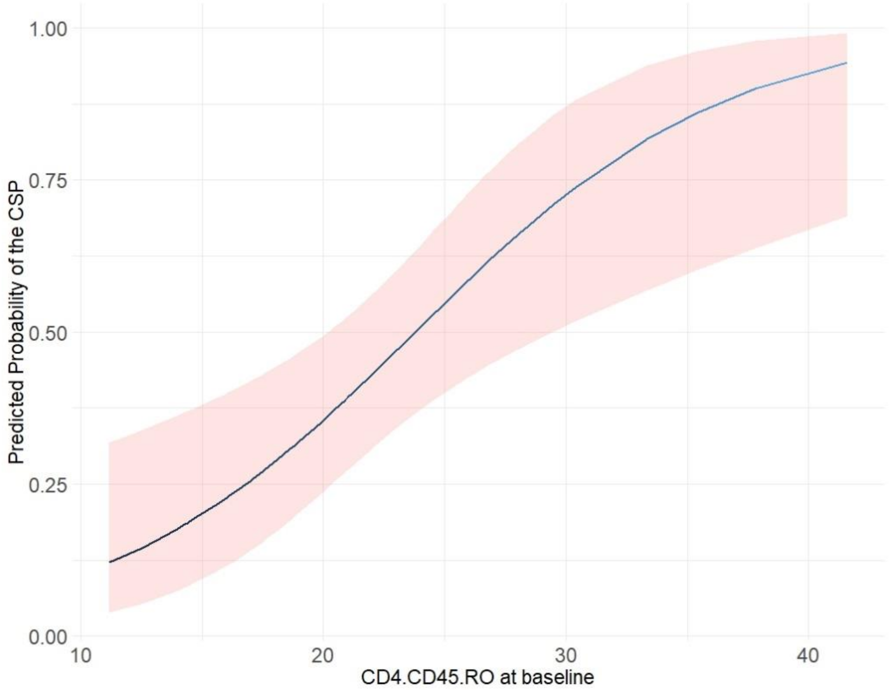

Supplementary Figure 5. Predicted probability of the CSP (CD4+/CD45RO+ab at baseline). Legend: The figure shows the predicted probability of the CSP (on vertical axis) for the range of measured values (on the horizontal axis) of a lymphocyte parameter (labelled at the bottom of the figure). The

blue curve depicts the point estimate of the probability and the pink area represents its 95% confidence interval.

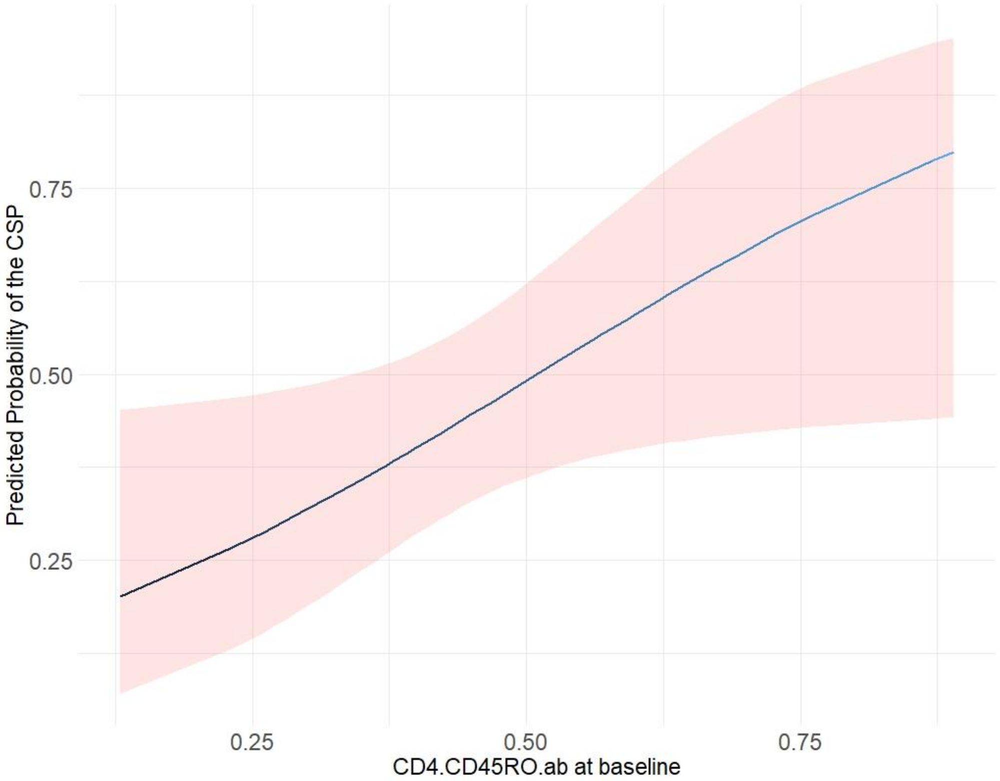

Supplementary Figure 6. Predicted probability of the CSP (CD8 at baseline). Legend: The figure shows the predicted probability of the CSP (on vertical axis) for the range of measured values (on the horizontal axis) of a lymphocyte parameter (labelled at the bottom of the figure). The blue curve depicts the point estimate of the probability and the pink area represents its 95% confidence interval.

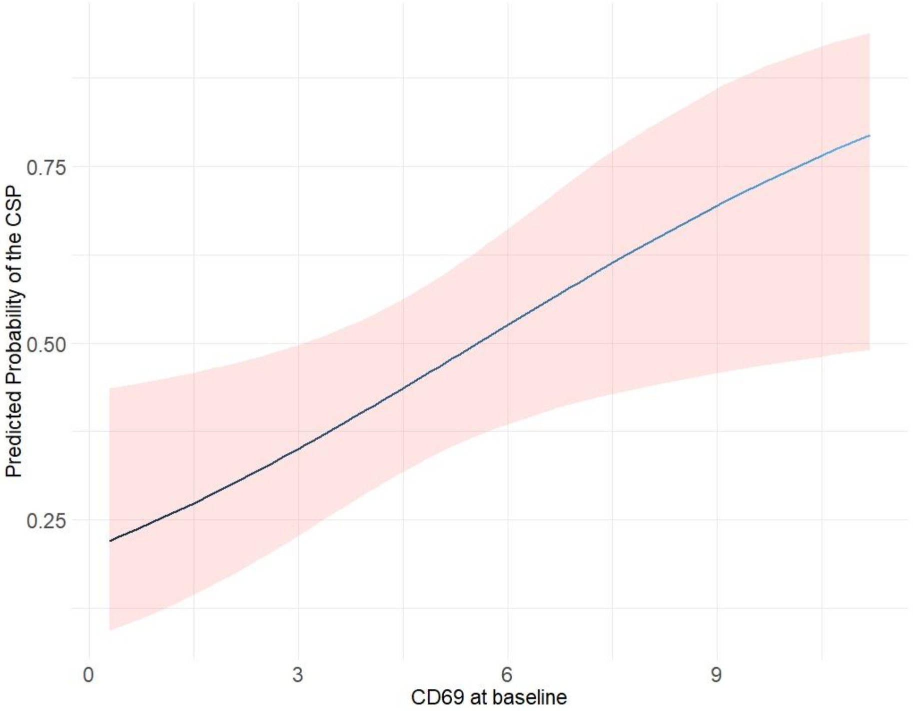

Supplementary Figure 7. Predicted probability of the CSP (CD69 at baseline). Legend: The figure shows the predicted probability of the CSP (on vertical axis) for the range of measured values (on the horizontal axis) of a lymphocyte parameter (labelled at the bottom of the figure). The blue curve depicts the point estimate of the probability and the pink area represents its 95% confidence interval.

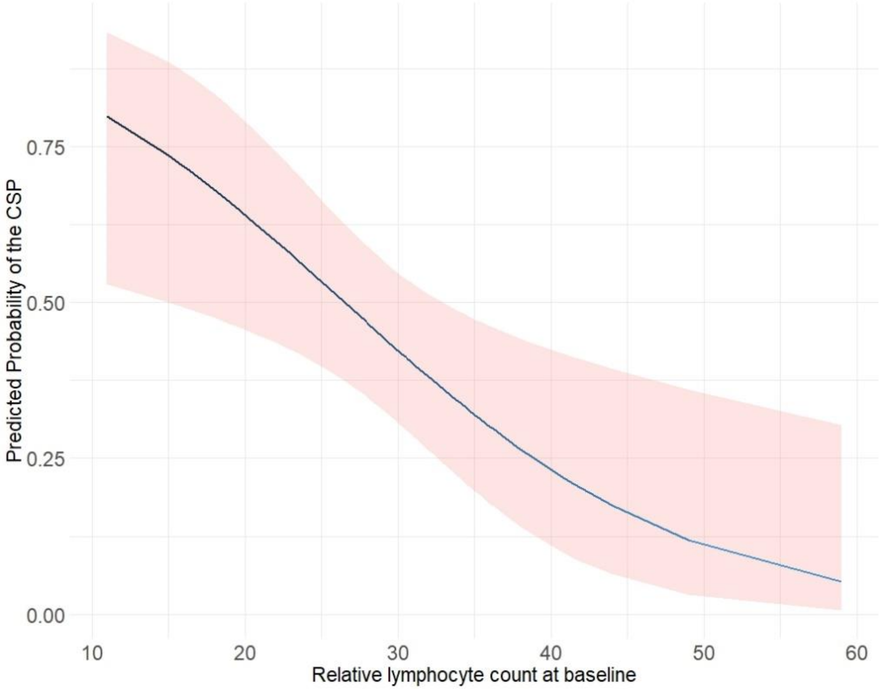

Supplementary Figure 8. Predicted probability of the CSP (relative lymphocyte count at baseline). Legend: The figure shows the predicted probability of the CSP (on vertical axis) for the range of measured values (on the horizontal axis) of a lymphocyte parameter (labelled at the bottom of the figure). The blue curve depicts the point estimate of the probability and the pink area represents its 95% confidence interval.

Supplementary Table 1. Antibodies, their provenience and dilution.

specifity	clone	fluorochrome	amount of undiluted antibody use in test	manufacturer
anti-CD3	UCHT1	FITC	10 µl	Beckman Coulter
anti-CD4	13B8.2	FITC	10 µl	Beckman Coulter
anti-CD45RA	ALB11	FITC	10 µl	Beckman Coulter
anti-CD8	B9.11	FITC	10 µl	Beckman Coulter
anti-CD19	J3-119	FITC	10 µl	Beckman Coulter
anti-CD25	B1.49.9	PE	10 µl	Beckman Coulter
anti-CD69	TP1.55.3	PE	10 µl	Beckman Coulter
anti-CD4	13B8.2	PE	10 µl	Beckman Coulter

anti-CD45RO	UCHL1	PE	10 µl	Beckman Coulter
anti-CD38	LS198-4-3	PE	10 µl	Beckman Coulter
anti-CD5	BL1a	PE	10 µl	Beckman Coulter
anti-CD40	MAB89	PE	10 µl	Beckman Coulter
anti-CD40L	TRAP-1	PE	10 µl	Beckman Coulter

Suppplementary Table 2. Descriptive characteristics for all 37 parameters and EDSS groups' comparison.

Characteristic		All (72)	CSP (32)	Non SP (40)	Groups' comparison	
					p value	Effect Size
absolute lymphocyte count	Baseline	2.04±0.68	1.96±0.69	2.11±0.66	0.353	0.22
	5 Years	2.2±0.64	1.99±0.66	2.36±0.59	0.016	0.59
	abs.	0.16±0.76	0.04±0.73	0.25±0.78	0.233	0.29
	rel. (%)	15.99±44.87	9.93±40.07	20.84±48.32	0.245	0.24
CD3	Baseline	76.27±7.19	77.92±7.09	74.96±7.08	0.073	0.42
	5 Years	76.15±7.02	74.94±6.88	77.13±7.06	0.19	0.31
	abs.	-0.12±7.45	-2.98±7.97	2.17±6.20	0.004	0.73
	rel. (%)	0.35±9.85	-3.29±10.15	3.27±8.67	0.004	0.7
CD3ab	Baseline	1.56±0.56	1.52±0.55	1.59±0.57	0.625	0.12
	5 Years	1.69±0.55	1.50±0.52	1.83±0.54	0.01	0.63
	abs.	0.13±0.62	-0.02±0.59	0.25±0.62	0.066	0.44
	rel. (%)	16.15±43.37	6.58±39.83	23.81±45.02	0.058	0.4
CD4	Baseline	48.29±8.67	48.77±7.05	47.90±9.85	0.673	0.1
	5 Years	49.21±8.24	49.88±6.78	48.68±9.30	0.544	0.14
	abs.	0.92±6.8	1.10±8.06	0.78±5.70	0.725	0.05
	rel. (%)	3.33±15.04	3.79±17.49	2.96±12.98	0.818	0.05
CD4ab	Baseline	0.99±0.41	0.95±0.35	1.02±0.46	0.475	0.17
	5 Years	1.09±0.38	1.00±0.36	1.16±0.39	0.086	0.41
	abs.	0.1±0.38	0.05±0.36	0.13±0.40	0.349	0.22
	rel. (%)	18.29±40.2	13.08±39.43	22.47±40.81	0.328	0.23
CD8	Baseline	26.9±6.52	28.54±7.50	25.59±5.36	0.055	0.46
	5 Years	25.51±5.93	24.21±4.96	26.55±6.48	0.096	0.4
	abs.	-1.39±6.52	-4.33±6.93	0.97±5.14	<0.001	0.88
	rel. (%)	-2.53±22.5	-11.74±20.64	4.84±21.38	0.001	0.79
CD8ab	Baseline	0.54±0.21	0.55±0.23	0.53±0.18	0.688	0.1
	5 Years	0.56±0.22	0.48±0.19	0.63±0.22	0.003	0.72
	abs.	0.02±0.27	-0.07±0.25	0.09±0.27	0.008	0.65
	rel. (%)	15.58±60.39	-2.40±43.30	29.97±68.35	0.02	0.55
CD19	Baseline	9.75±4.44	8.44±3.90	10.79±4.62	0.014	0.54

	5 Years	10.66±3.94	10.77±3.63	10.58±4.21	0.844	0.05
	abs.	0.83±3.89	2.29±3.27	-0.33±3.99	0.008	0.71
	rel. (%)	66.3±395.34	139.88±589.39	8.21±53.76	0.013	0.34
CD19ab	Baseline	0.2±0.11	0.18±0.11	0.22±0.10	0.056	0.41
	5 Years	0.24±0.12	0.22±0.12	0.25±0.11	0.216	0.23
	abs.	0.03±0.12	0.04±0.13	0.02±0.12	0.466	0.18
	rel. (%)	36.58±86.87	48.56±111.47	27.43±62.12	0.604	0.24
natural killer	Baseline	11.52±6.9	11.29±7.15	11.71±6.79	0.706	0.06
	5 Years	10.6±5.27	11.34±5.95	10.01±4.64	0.458	0.25
	abs.	-0.93±6.31	0.36±7.25	-1.96±5.33	0.182	0.37
	rel. (%)	16.38±78.35	28.41±86.37	6.89±71.12	0.246	0.28
natural killer ab	Baseline	0.23±0.15	0.22±0.14	0.24±0.16	0.52	0.15
	5 Years	0.22±0.12	0.22±0.12	0.23±0.11	0.618	0.1
	abs.	-0.01±0.15	0.00±0.14	-0.03±0.16	0.753	0.18
	rel. (%)	34.89±133.91	34.33±108.05	35.33±152.72	0.892	0.01
absolute total leukocyte count	Baseline	7.19±2.3	7.80±2.86	6.70±1.62	0.141	0.49
	5 Years	7.23±1.67	7.03±1.83	7.39±1.53	0.319	0.21
	abs.	0.04±2.35	-0.77±2.69	0.69±1.84	0.007	0.64
	rel. (%)	7.12±31.91	-2.34±32.18	14.69±29.98	0.023	0.55
relative lymphocyte count	Baseline	29.45±8.68	26.28±7.91	31.98±8.52	0.005	0.69
	5 Years	30.94±7.78	29.16±8.52	32.37±6.92	0.082	0.42
	abs.	1.49±9.9	2.88±11.31	0.39±8.60	0.292	0.25
	rel. (%)	13.27±44.27	22.65±56.16	5.77±30.52	0.421	0.39
CD3+/CD69+	Baseline	2.84±2.26	3.53±2.56	2.28±1.83	0.035	0.57
	5 Years	1.38±0.61	1.35±0.62	1.40±0.61	0.597	0.08
	abs.	-1.46±2.31	-2.22±2.56	-0.86±1.92	0.022	0.61
	rel. (%)	-4.46±121.6	-16.50±118.54	5.04±124.71	0.034	0.18
CD5	Baseline	76.8±7.95	78.12±8.24	75.76±7.67	0.227	0.3
	5 Years	75.6±10.91	75.81±6.53	75.42±13.52	0.402	0.04
	abs.	-1.15±12.26	-2.52±8.70	-0.07±14.49	0.029	0.2
	rel. (%)	-0.69±15.68	-2.36±11.59	0.63±18.32	0.026	0.19
CD40 ligand	Baseline	0.34±0.57	0.41±0.67	0.28±0.49	0.169	0.23
	5 Years	0.39±1.53	0.20±0.17	0.55±2.05	0.432	0.23
	abs.	-0.13±0.61	-0.21±0.65	-0.06±0.57	0.216	0.26
	rel. (%)	24.76±185.22	-5.74±113.36	53.98±233.44	0.7	0.32
CD25	Baseline	6.33±3.94	7.25±4.62	5.61±3.20	0.246	0.42
	5 Years	5.55±2.25	5.70±2.47	5.43±2.08	0.627	0.12
	abs.	-0.78±4.77	-1.59±5.00	-0.14±4.55	0.426	0.3

	rel. (%)	48.04±180.84	22.53±125.92	68.18±214.11	0.299	0.25
CD38	Baseline	53.63±12.92	51.06±15.98	55.52±9.91	0.608	0.35
	5 Years	47.96±9.36	46.33±8.62	49.27±9.82	0.187	0.32
	abs.	-5.35±13.59	-4.82±16.88	-5.74±10.77	0.787	0.07
	rel. (%)	4.35±96.52	21.64±145.51	-8.39±22.88	0.795	0.31
CD40	Baseline	9.43±4.34	8.51±3.96	10.15±4.53	0.124	0.38
	5 Years	10.55±4.44	10.99±4.06	10.19±4.74	0.45	0.18
	abs.	1.12±3.9	2.31±3.98	0.19±3.61	0.025	0.56
	rel. (%)	55.59±260.71	103.92±382.04	17.43±72.75	0.029	0.33
CD3+/CD25+	Baseline	5.73±3.63	6.54±4.27	5.09±2.93	0.238	0.4
	5 Years	5.23±2.06	5.33±2.34	5.14±1.84	0.715	0.09
	abs.	-0.5±4.3	-1.24±4.65	0.09±3.97	0.447	0.31
	rel. (%)	60.15±204.32	31.92±140.84	82.44±242.68	0.374	0.25
CD69	Baseline	5.48±7.23	5.57±3.03	5.40±9.36	0.041	0.02
	5 Years	3.03±1.69	2.90±1.32	3.13±1.94	0.86	0.14
	abs.	-2.46±7.5	-2.73±3.33	-2.25±9.64	0.027	0.06
	rel. (%)	5.69±135.78	-3.65±156.90	13.07±118.15	0.063	0.12
CD5+/CD19+	Baseline	1.6±1.69	1.19±1.11	1.93±1.99	0.09	0.44
	5 Years	1.55±1.05	1.48±0.73	1.61±1.26	0.7	0.12
	abs.	-0.06±1.3	0.23±0.94	-0.29±1.50	0.172	0.41
	rel. (%)	95.13±262.97	134.61±246.38	64.19±274.59	0.079	0.27
CD4+/CD45RO+	Baseline	22.2±6.37	25.13±5.88	19.88±5.83	<0.001	0.9
	5 Years	23.79±6.11	26.45±5.39	21.66±5.87	0.001	0.84
	abs.	1.46±5.21	1.33±6.15	1.56±4.42	0.86	0.04
	rel. (%)	10.05±25.53	8.74±25.75	11.09±25.65	0.709	0.09
CD4+/CD45RA+	Baseline	19.72±8.06	17.37±7.08	21.57±8.39	0.056	0.54
	5 Years	21.33±8.76	20.16±9.06	22.27±8.51	0.312	0.24
	abs.	1.96±5.18	3.00±5.20	1.14±5.08	0.121	0.36
	rel. (%)	14.82±39.95	21.43±50.70	9.61±28.49	0.148	0.3
CD8+/CD38+	Baseline	12.05±6.42	13.14±7.39	11.19±5.48	0.217	0.3
	5 Years	9.03±4.66	8.24±4.12	9.66±5.01	0.142	0.31
	abs.	-2.95±7	-4.89±8.41	-1.42±5.29	0.042	0.51
	rel. (%)	11.67±123.34	-3.08±109.78	23.32±133.36	0.064	0.21
CD3+/CD69+ab	Baseline	0.06±0.06	0.07±0.05	0.05±0.06	0.043	0.32
	5 Years	0.03±0.02	0.03±0.01	0.03±0.02	0.061	0.41
	abs.	-0.03±0.06	-0.05±0.05	-0.02±0.06	0.004	0.43

	rel. (%)	-11.63±78.81	-39.76±59.21	10.42±85.74	0.003	0.67
CD5ab	Baseline	1.58±0.57	1.56±0.56	1.60±0.59	0.946	0.06
	5 Years	1.66±0.56	1.52±0.54	1.76±0.56	0.067	0.44
	abs.	0.07±0.69	-0.04±0.61	0.16±0.74	0.226	0.3
	rel. (%)	13.71±46.57	5.37±40.85	20.29±50.19	0.091	0.32
CD40 ligand+ab	Baseline	0.01±0.02	0.01±0.02	0.01±0.01	0.534	0.15
	5 Years	0.01±0.03	0.00±0.00	0.01±0.04	0.364	0.26
	abs.	0±0.02	-0.00±0.02	-0.00±0.02	0.161	0.23
	rel. (%)	-87.13±25.69	-77.04±33.23	-97.22±8.33	0.065	0.83
CD25ab	Baseline	0.13±0.1	0.15±0.12	0.11±0.07	0.38	0.39
	5 Years	0.12±0.06	0.11±0.07	0.13±0.05	0.11	0.2
	abs.	-0.01±0.1	-0.04±0.10	0.01±0.10	0.043	0.5
	rel. (%)	67.63±256.31	25.78±114.45	100.67±325.81	0.137	0.29
CD38ab	Baseline	1.09±0.49	0.97±0.43	1.18±0.52	0.197	0.43
	5 Years	1.06±0.38	0.92±0.33	1.16±0.39	0.005	0.68
	abs.	-0.03±0.51	-0.06±0.45	-0.01±0.56	0.662	0.11
	rel. (%)	22.25±124.5	37.88±182.27	10.74±51.61	0.467	0.22
CD40ab	Baseline	0.2±0.11	0.18±0.11	0.21±0.11	0.146	0.29
	5 Years	0.24±0.13	0.23±0.15	0.24±0.12	0.401	0.08
	abs.	0.04±0.14	0.05±0.15	0.03±0.13	0.549	0.15
	rel. (%)	43.72±106.23	45.87±100.52	42.08±111.70	0.752	0.04
CD3+/CD25+ab	Baseline	0.12±0.09	0.14±0.11	0.10±0.07	0.279	0.37
	5 Years	0.11±0.06	0.11±0.07	0.12±0.05	0.065	0.23
	abs.	0±0.09	-0.03±0.09	0.01±0.09	0.042	0.51
	rel. (%)	79.36±260.27	43.96±177.17	107.31±310.24	0.1	0.24
CD69ab	Baseline	0.11±0.1	0.11±0.07	0.10±0.13	0.151	0.05
	5 Years	0.06±0.03	0.06±0.03	0.07±0.03	0.029	0.42
	abs.	-0.04±0.11	-0.05±0.07	-0.03±0.13	0.018	0.18
	rel. (%)	6.11±95.22	-18.34±76.50	25.42±104.71	0.034	0.47
CD5+/CD19+ab	Baseline	0.03±0.03	0.02±0.02	0.04±0.04	0.112	0.41
	5 Years	0.03±0.03	0.03±0.02	0.04±0.03	0.678	0.23
	abs.	0±0.03	0.01±0.02	0.00±0.04	0.688	0.16
	rel. (%)	31.34±92.57	36.03±74.32	27.89±104.95	0.297	0.09
CD4+/CD45RO+ab	Baseline	0.44±0.15	0.49±0.16	0.41±0.14	0.01	0.54
	5 Years	0.51±0.18	0.52±0.18	0.51±0.19	0.586	0.05
	abs.	0.07±0.19	0.03±0.18	0.09±0.19	0.203	0.31

	rel. (%)	24.2±50.74	13.33±40.26	32.78±56.74	0.117	0.39
CD4+/CD45RA+ab	Baseline	0.41±0.24	0.36±0.19	0.46±0.26	0.073	0.44
	5 Years	0.48±0.27	0.41±0.24	0.54±0.27	0.044	0.49
	abs.	0.08±0.18	0.06±0.17	0.09±0.19	0.444	0.16
	rel. (%)	29.24±57.04	28.43±65.19	29.88±50.59	0.574	0.03
CD8+/CD38+ab	Baseline	0.25±0.16	0.26±0.16	0.24±0.15	0.455	0.17
	5 Years	0.2±0.12	0.16±0.08	0.23±0.13	0.003	0.63
	abs.	-0.05±0.2	-0.11±0.18	-0.01±0.20	0.036	0.52
	rel. (%)	29.17±150.55	1.66±128.82	50.88±164.13	0.024	0.33

Legend: The reported statistics are mean ±standard deviation. Effect size is assessed by Cohen's D. All P values are reported after Benjamini-Hochberg correction.

Supplementary Table 3. Univariate logistic regression models for values of lymphocytes' parameters in the fifth year and their changes (absolute and relative) and multivariate ones with adjustment by covariates: EDSS, age and gender.

Variable	Univariate logistic regression					Multivariate logistic regression with covariates				
	OR	95% LCI OR	95% UCI OR	*p*	R2	OR	95% LCI OR	95% UCI OR	*p*	R2
absolute.lymphocyte.count.5	0.37	0.15	0.82	0.0213	0.11	0.36	0.14	0.81	0.0202	0.13
CD3ab.5	0.28	0.09	0.73	0.0152	0.12	0.28	0.09	0.74	0.0161	0.14
CD8ab.5	0.02	0	0.29	0.0066	0.16	0.02	0	0.28	0.0064	0.18
CD4+/CD45RO+..5	1.16	1.06	1.29	0.002	0.2	1.18	1.08	1.32	0.0011	0.25
CD38ab.5	0.13	0.02	0.54	0.009	0.15	0.13	0.02	0.56	0.01	0.16
CD8+/CD38+..ab.5	0	0	0.15	0.015	0.15	0	0	0.22	0.0225	0.16

CD3.abs	0.9	0.82	0.97	0.0111	0.17	0.9	0.82	0.97	0.0121	0.18
CD8.abs	0.86	0.77	0.94	0.0025	0.23	0.85	0.76	0.94	0.0024	0.26
CD8ab.abs	0.07	0.01	0.5	0.0132	0.13	0.05	0	0.41	0.0095	0.17
CD19.abs	1.24	1.06	1.49	0.0118	0.25	1.23	1.04	1.49	0.0233	0.26
absolute leukocyte count.abs	0.76	0.57	0.98	0.0446	0.14	0.76	0.56	0.98	0.0481	0.16
CD3+/CD69+..abs	0.76	0.59	0.95	0.0214	0.2	0.75	0.58	0.95	0.0213	0.24
CD5.abs	0.91	0.84	0.98	0.0167	0.24	0.91	0.84	0.98	0.0249	0.25
CD40.abs	1.16	1.02	1.35	0.0308	0.19	1.15	1	1.34	0.0687	0.2
CD69.abs	0.8	0.65	0.95	0.0147	0.24	0.8	0.66	0.95	0.0195	0.27
CD3+/CD69+..ab.abs	0	0	0	0.0081	0.32	0	0	0	0.008	0.34
CD8+/CD38+..ab.abs	0.01	0	0.39	0.0191	0.25	0.02	0	0.47	0.026	0.27
CD3.rel	0.92	0.86	0.98	0.0088	0.15	0.92	0.86	0.98	0.0099	0.17
CD8.rel	0.96	0.93	0.98	0.0035	0.19	0.96	0.93	0.98	0.003	0.22
CD8ab.rel	0.99	0.98	1	0.0641	0.11	0.99	0.97	1	0.0409	0.15
CD19.rel	1.02	1.01	1.04	0.012	0.31	1.02	1	1.04	0.0158	0.33
Absolute total leukocyte count rel	0.98	0.96	1	0.0455	0.1	0.98	0.96	1	0.054	0.12

CD3+/CD69+..rel	0.99	0.98	1	0.0389	0.27	0.99	0.98	1	0.0485	0.3
CD5.rel	0.94	0.88	0.99	0.0284	0.22	0.94	0.88	0.99	0.0415	0.24
CD40.rel	1.01	1	1.03	0.0346	0.29	1.01	1	1.03	0.0615	0.31
CD3+/CD69+..ab.rel	0.98	0.97	0.99	0.0058	0.4	0.98	0.97	0.99	0.0081	0.43

Legend: OR - Odds ratio, 95% LCI OR is lower 95% confidence limit of OR, 95% UCI is upper 95% confidence limit of OR, R2 is Nagelkerke's pseudo R squared that assesses the quality of fit (the higher, the better). Suffix (in the name of variable) .5 is value at the 5th year, .abs and .rel are absolute and relative changes after 5 years.
